# Observations on Twin Cases

**Published:** 1811-10

**Authors:** 


### *To the Editors of the Medical and Physical Journal.*

Gentlemen,

Allow me to say a few words in reply to Mr. Fogo's Remarks on the Case of Twins which I sent for publication in your Journal for June last. He begins by a misstatement in saying that I called myself an "inexperienced practitioner." I acknowledged myself a young man, and that confession surely ought to have disarmed criticism of any unusual severity. It was the first case in which I had attended the delivery of twins; I never before delivered a fœtus with the forceps, nor had my hand till then been introduced into a uterus for the purpose of turning and delivering by the feet. I was alone; no medical man resided within five miles of me, and the only resource left me was my own immatured judgment, and Denman's Introduction to Midwifery which I had taken with me. "I condemned myself for rashness" because Denman writes as follows: 'Without regard to those who are fond of speculative opinions, or the determination of those who are guided by practice alone, I have concluded that we may safely, and ought to wait *four hours at least* after the birth of the first child, before we deliver a patient *by art* of a second child, if there be no particular cause for delivering her sooner; by this decision we shall avoid many unnecessary operations without detriment to the patient, without increasing our own difficulties, or hazarding our reputation.' And, further, I was not convinced that any particular symptom rendered immediate interference necessary. The woman was very languid and low before labour-pains first came on; some circumstances made her situation peculiarly distressing; herself and friends were placed in a middling station of life, and she had been seduced under a promise of marriage, and, at an advanced period of pregnancy, arrangements were concluded for the celebration of the nuptials; even a dinner for the occasion was prepared, and the friends of the young woman had assembled in order to accompany her to church; at this moment the young man receded from his engagements, and left the poor girl a prey to wretchedness and remorse. Surely, then, such a peculiarity of situation would account for "great languor" and dejection of spirits without any other cause; and was that sufficient ground for the immediate delivery of the second child? I waited two hours after delivering the first, "during that time (says Mr. Fogo) the woman was losing blood; but being prevented prevented from passing the os externum by the placenta of the first, and by the body, membranes, and waters of the second child, it did not, nor could not, make its appearance." He also takes it for granted, that during this period a great pool of blood was accumulating from the detachment of the first placenta, which was confined, as before mentioned, till a passage was opened for it. Now had this really been the case, I must have met with these obstructing causes on introducing my hand; on the contrary, neither the placenta, membranes or coagula were to be felt either at the os externum or the os uteri; that part of the uterus which had contained the child delivered was unoccupied, at least so much so, that my fingers passed some distance from the os uteri before they were opposed by the membranes of the second fœtus; the placenta which Mr. F. has so conveniently placed at the os uteri was not to be found. I traced the chord till it was lost behind the membranes, enveloping the remaining fœtus. The entire absence of pains during the delivery of the first child, and subsequent to it, accounts for the uterus being in this state; it appeared to have lost its usual powers of contracting; one or both placentæ adhered to the uterus, which rendered separation with my fingers necessary. I cannot now state whether they were united; this is an omission for which regret is now useless. The hæmorrhage was truly such as I had never witnessed. I stated the loss of blood was six or seven pounds; I spoke within bounds: the expression "in a few moments," I admit was indefinite and vague; it was thrown out with great rapidity, and continued to flow till she sunk without any appearance of life. One of the children is now alive, the other lived several months. I pretended not (as Mr. Fogo insinuates) to the knowledge of discovering a twin case prior to the birth of the first child; surely there was no vanity in stating that the child which I had delivered with the forceps being a little undersize, connected with the large appearance of the woman, led me to judge there were twins. I can also assure Mr. Fogo that I have attended a respectable teacher, (Dr. Haighton,) and that I have also read respectable books on the subject of midwifery. As I advance in years I hope to add to my small share of knowledge; his criticisms will, perhaps, stimulate my exertions, but at present they fail to convince me, that I delivered the woman "but just in time to save her life," or that if I had delivered her two hours sooner I should have been justified by both "Theory and Practice."

I am, Gentlemen,

Your most obedient servant,

AN ESSEX PRACTITIONER.

*August* 10*th*, 1811.

*P. S.* I shall expect, with impatience, Mr. Fogo's Observations on the "Retrograde" State of the Obstetric Art, and on the "Absurdity of Guarding the Perineum." If it is really necessary for us to "unlearn" that which has cost most of us much expence and labour to acquire, the sooner we begin the better.

